# Landscape genetic inferences vary with sampling scenario for a pond‐breeding amphibian

**DOI:** 10.1002/ece3.5023

**Published:** 2019-04-15

**Authors:** Travis Seaborn, Samantha S. Hauser, Lauren Konrade, Lisette P. Waits, Caren S. Goldberg

**Affiliations:** ^1^ School of Biological Sciences Washington State University Pullman Washington; ^2^ Department of Biology University of Louisiana Lafayette Louisiana; ^3^ Department of Biological Sciences Wichita State University Wichita Kansas; ^4^ Department of Fish and Wildlife Sciences University of Idaho Moscow Idaho; ^5^ School of the Environment Washington State University Pullman Washington

**Keywords:** amphibian ecology, Circuitscape, Columbia spotted frog, connectivity, landscape genetics, sampling schemes

## Abstract

A critical decision in landscape genetic studies is whether to use individuals or populations as the sampling unit. This decision affects the time and cost of sampling and may affect ecological inference. We analyzed 334 Columbia spotted frogs at 8 microsatellite loci across 40 sites in northern Idaho to determine how inferences from landscape genetic analyses would vary with sampling design. At all sites, we compared a proportion available sampling scheme (PASS), in which all samples were used, to resampled datasets of 2–11 individuals. Additionally, we compared a population sampling scheme (PSS) to an individual sampling scheme (ISS) at 18 sites with sufficient sample size. We applied an information theoretic approach with both restricted maximum likelihood and maximum likelihood estimation to evaluate competing landscape resistance hypotheses. We found that PSS supported low‐density forest when restricted maximum likelihood was used, but a combination model of most variables when maximum likelihood was used. We also saw variations when AIC was used compared to BIC. ISS supported this model as well as additional models when testing hypotheses of land cover types that create the greatest resistance to gene flow for Columbia spotted frogs. Increased sampling density and study extent, seen by comparing PSS to PASS, showed a change in model support. As number of individuals increased, model support converged at 7–9 individuals for ISS to PSS. ISS may be useful to increase study extent and sampling density, but may lack power to provide strong support for the correct model with microsatellite datasets. Our results highlight the importance of additional research on sampling design effects on landscape genetics inference.

## 1 INTRODUCTION

Habitat loss and fragmentation is one of the largest threats to wildlife populations worldwide. As global landscape change continues to accelerate, there is an increasing need to understand how species respond (Cushman, 2006). Knowledge of movement ecology and connectivity is difficult to obtain for many species, but is essential for evaluating population viability of a species at the regional scale (Fahrig & Merriam, 1994). Animal movement is often studied by physical tracking, which has a rich history of use across taxa with a variety of methodologies (Aarts, MacKenzie, McConnell, Fedak, & Matthiopoloulos, 2008; Langkilde & Alford, 2002); however, because movement does not always indicate the transfer of genes (Semlitsch, 2008), it alone is not the most appropriate tool for measuring functional connectivity.

Landscape genetics combines landscape ecology and population genetics to evaluate functional connectivity, which provides inferences about factors affecting movement and reproduction (Manel, Schwartz, Luikart, & Taberlet, 2003, Holderegger & Wagner, 2006, Manel & Holderegger 2013). Quantitative methods that link landscape features and genetic data allow researchers to infer migration events between populations (Storfer *et al*. 2007). By collecting genetic data across a landscape, researchers can identify how spatial genetic patterns may be influenced by landscape features (Manel et al., 2003). For example, common genetic patterns include isolation by distance (IBD; Wright, 1943), barriers to movement (IBB; Cushman, 2006), isolation by environment (IBE; Wang & Bradburd, 2014), and isolation by resistance (IBR; McRae, 2006).

Landscape genetic sampling schemes can be difficult to properly develop, identify, and implement (Manel et al., 2003; Oyler‐McCance, Fedy, & Landguth, 2013; Segelbacher et al., 2010). The sampling scheme used in landscape genetics studies depends on the distribution of the species, the spatial and temporal scales of processes of interest, and availability of resources allocated to sampling (Balkenhol, Cushman, Storfer, & Waits, 2015; Manel et al., 2003; Schwartz & McKelvey, 2008). Inefficient or biased sampling design can decrease the ability of a study to correctly identify the processes leading to population structure (Oyler‐McCance et al., 2013). Sampling schemes need to consider the extent of the study area and the distance between potential sampling sites, as well as species distribution, temporal scale, and life history traits of the study organism (Anderson et al., 2010; Prunier et al., 2013). There are two broad groups of study design sampling types, using either individual or population as the unit of analysis, which often overlap in their hypotheses but vary in their overall approaches (Dyer, 2015). With individual sampling scheme (ISS), only one or few individuals are sampled per geographic location and genetic distances are calculated between all pairs of individuals to create matrices based on individual genotypes (Coulon et al., 2004; Prunier et al., 2013). In contrast, sampling at the population level can be applied where ecologically relevant population delineations occur by sampling many individuals in each aggregate and creating distance matrices by either averaging interindividual distance matrices, as we have done here, or by using population‐level genetic distances, for example, *F*_ST_ (Spear, Peterson, Matocq, & Storfer, 2005). The population‐level sampling scheme (PSS) can be problematic because populations are often difficult to delineate a priori, and sufficient sample sizes of many species are difficult to obtain (Manel et al., 2003). A PSS is resource‐ and time‐consuming and often results in a reduced sampling extent or a more diffuse sampling regime, leaving areas unsampled (Prunier et al., 2013). If fewer than the target number of individuals is collected at a location, that population is often dropped from the final analysis, leaving a gap in sampling and excluding potentially informative genetic data. In addition, a PSS may not be appropriate for species where population delineation is difficult or habitat use is continuous, like highly mobile or migratory species. In these continuous distribution systems, ISS may be most appropriate (Luximon, Petit, & Broquet, 2014).

Despite the drawbacks of the PSS, it is more commonly utilized than ISS (Prunier et al., 2013) because of well‐developed population genetic theory and analysis. There is a third, unexplored option, which is to include all individuals from all populations, regardless of number of individuals sampled from each population, which we refer to as proportion available sampling scheme (PASS). This sampling scheme could be utilized when a target number of individuals are not obtained at each sampling area due to low densities, time, funding, or other constraints. Here, we aim to compare the ability of landscape genetic analyses to detect landscape genetic patterns using these three alternative sampling schemes (Box 1).

BOX 1 **Glossary of sampling scheme terms and abbreviations**

1

**Table:** 

Scheme	Abbreviation	Definition	Example indices
Individual Level Sampling Scheme	ISS	One or few individuals sampled per population. All sampled populations included in analysis.	Proportion of Shared Alleles, Bray‐Curtis Dissimilarity
Population Level Sampling Scheme	PSS	Many individuals sampled in aggregate, minimum number of (often 20 or more) individuals required to include population in analysis.	Average Proportion of Shared Alleles, Nei's Da, Fst
Proportion Available Sampling Scheme	PASS	Include all individuals and populations sampled in analysis, regardless of number of individuals per population sampled.	Average Proportion of Shared Alleles

To understand how inference of landscape genetic patterns can differ based on sampling scheme, we studied a pond‐breeding species, the Columbia spotted frog (*Rana luteiventris*) in northern Idaho, USA, over an area of 1,555 km^2^. The Columbia spotted frog is a wide‐ranging species, with a distribution from the southern Rocky Mountains to southeastern Alaska (Green, Kaiser, Sharbel, Kearsley, & McAllister, 1997). In northern Idaho, breeding populations are often small, with effective population sizes ranging from 3.2 to 37.8; because of this, the persistence of the Columbia spotted frogs in the region may be at risk (Davis & Verrell, 2005; Goldberg & Waits, 2009). Within a smaller extent in this area of the range (213 km^2^), Columbia spotted frog functional connectivity was found to be negatively influenced by forest presence, while shrub/clear‐cut and agriculture land cover types were found to have the lowest resistance to gene flow (Goldberg & Waits, 2010a). Pond‐breeding amphibians are a useful model to investigate sampling scheme questions because, due to their distribution and population sizes, they can be sampled using either the ISS or the PSS. Although PSS may be more appropriate for the Columbia spotted frog due to pond‐breeding amphibians being generally philopatric (Smith & Green, 2005), this system allowed us to evaluate sampling schemes ranging from a single individual to the population level in an iterative manner. We compared the level of functional connectivity inferred by individual, population, and proportion available sampling schemes using an information theoretic approach to model landscape resistance (Burnham & Anderson, 2002). The candidate models consisted of slope, solar radiation, and the following land cover types: water, high‐density forest, low‐density forest, agriculture, shrub, grassland, and human development in varying combinations.

Our objective was to compare landscape genetic inferences of connectivity (a) among individual and population schemes, and (b) at different sampling densities. The increase in sampling densities, the number of sample locations within a given area, also corresponded to a slightly increased extent, representing a probable scenario if a PASS was implemented in a new system. Although this increase in area occurs by adding populations only 5–15 km away from existing sites, this represents a biologically meaningful increase to a species where adult migration is <2 km on average (Bull & Hayes, 2001; Pilliod, Peterson, & Ritson, 2002). We expected that ISS would indicate the same variables overall as PSS, albeit with less support. We used multiple random draws at locations with more than one individual to create resampled ISS replicates. We expected that the ISS approach would indicate the same variables as PASS, but that including these sites with low sample sizes would add noise to the results; specifically, that multiple models would be supported in many of the resampled replicates. With higher numbers of individuals per site, we expected that the results would converge with the PSS based on the added statistical precision provided by more individuals. We did not have an expectation on the number of supported models or model weights across all datasets and replicates.

## 2 MATERIALS AND METHODS

The study area (Latah County, Idaho, USA) included two ecoregions (Palouse Prairie [West] and Bitterroot Mountains [East]) and their ecotone. The landscape has been largely altered through agriculture, human development, and forest management (Black et al., 1998; Dahl *et al*. 2000). The population size of the nearest city to the study area, Moscow, Idaho, increased 5.3% from 2010 to 2015, which is greater than the national average of 4.1% (United States Census Bureau, 2015). Only a small fraction (13%) of natural wetlands existed as of the most recent comprehensive survey (Black et al., 1998) posing potential limitations for amphibian populations.

**Figure 1:**
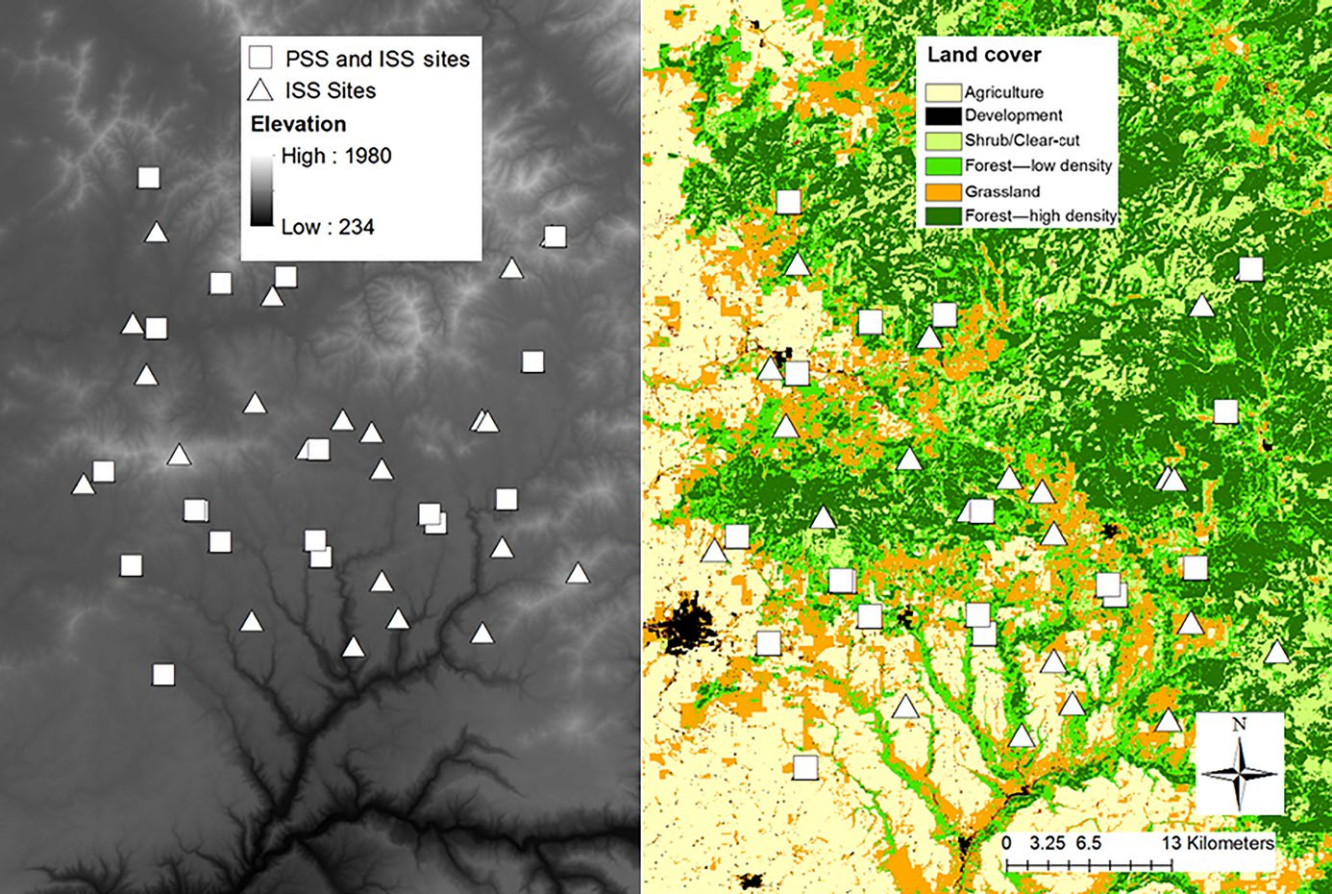
Elevation (left, meters) and land cover type (right) for wetland sampling locations for the Columbia spotted frog (*Rana luteiventris*) in northern Idaho (Idaho Geospatial Office, 2001). Population‐level sampling scheme (PSS) is locations where at least 11 individuals were sampled. Locations where population sampling occurred had one individual randomly selected for individual‐level sampling scheme replicate dataset (ISS)

We analyzed tissue samples (mouth swabs and tail clips) from 334 individuals sampled at 40 wetlands from the randomly selected set surveyed for habitat modeling in the study area (Goldberg & Waits, 2009; Figure 1). We extracted DNA from these samples using the DNeasy Blood and Tissue Kit (Qiagen). Samples were scored at eight nuclear DNA microsatellite loci with GENEMAPPER (Applied Biosystems, Inc.): Rp3, Rp15, Rp17, Rp23, SFC128, SFC134, SFC139, and RP193 (Monsen & Blouin, 2003, Funk et al., 2005, see Goldberg & Waits, 2010a for PCR reaction descriptions, Table 1 for list of alleles and frequencies). For tadpole samples, we detected siblings using a cutoff value of 0.75 in COLONY within sampling sites (Jones & Wang, 2010) and included only one individual from each set (as recommended in Goldberg & Waits, 2010b). Seven percent of samples were run twice to check for genotyping errors or other inconsistencies; none were found. We considered sites with ≥11 individuals as population‐level samples (*N* = 18 out of 40 possible wetlands). This was based on the distribution of the sample sizes collected in the field, with 5 of the 18 population‐level sites having 11 individuals collected. Prior to the landscape genetic analysis, we measured population genetic statistics on population‐level samples and tested for Hardy–Weinberg equilibrium using GENALEX (Peakall & Smouse, 2012) and linkage disequilibrium with ARLEQUIN (Excoffier & Lischer, 2010). We analyzed data with ISS at two sampling densities, for a total of four sampling schemes: population‐level sampling (PSS), individual‐level sampling at the 18 sites for which there were population‐level data (ISS‐18), individual‐level sampling at all 40 sites (ISS‐40), and a PSS‐ISS hybrid in which all individuals at all 40 sites were analyzed (PASS). The PASS and ISS‐40 datasets encompassed a slightly larger extent (Figure 1), representing a probable scenario if sampling effort per site was reduced and more sites were included. To determine the minimum sampling density for ISS approaches to reach the same conclusions as PSS/PASS, we also bootstrapped resampled subsets of 2 through 11 individuals.

**Table 1:** Allele list and average frequency for each microsatellite locus across 40 sampling areas of Columbia spotted frogs (*Rana luteiventris*)

Locus	Allele/*n*	Average frequency	Locus	Allele/*n*	Average frequency
RP3	80	0.10	RP15	85	0.00
	90	0.05		90	0.01
	100	0.03		100	0.08
	110	0.24		105	0.01
	120	0.02		110	0.73
	130	0.20		120	0.17
	140	0.33	RP23	40	0.01
	150	0.03		80	0.00
	170	0.00		90	0.04
RP17	90	0.01		100	0.65
	100	0.10		110	0.13
	110	0.35		120	0.06
	120	0.34		130	0.11
	130	0.01	SFC128	80	0.00
	220	0.03		90	0.06
	240	0.02		100	0.09
	250	0.00		110	0.08
	260	0.04		120	0.46
	270	0.01		130	0.18
	280	0.02		140	0.11
	310	0.01		150	0.08
	360	0.02		160	0.00
	370	0.00		170	0.00
	420	0.01	SFC134	100	0.06
	430	0.01		110	0.36
	440	0.00		115	0.01
	450	0.01		120	0.49
	460	0.00		130	0.01
	490	0.01		150	0.00
	510	0.00		160	0.00
	70	0.00		90	0.01
	100	0.01	RP193	95	0.02
	110	0.26		100	0.02
SFC139	120	0.05		110	0.01
	130	0.06		115	0.13
	140	0.05		117	0.03
	150	0.03		120	0.08
	160	0.10		125	0.04
	170	0.10		127	0.03
	180	0.05		130	0.54
	190	0.05		140	0.09
	200	0.04		150	0.01
	210	0.13			
	220	0.02			
	230	0.03			
	240	0.01			
	250	0.00			

### 2.1 Genetic distances

We used proportion of shared alleles, *D*_ps,_ as our metric of genetic distance as it can be estimated with both population‐ and individual‐level sampling (Bowcock et al., 1994). To evaluate the effects of the choice of genetic distance dissimilarity index, we calculated genetic distance with *D*_ps_ and Bray–Curtis percentage dissimilarity with the individual sampling scheme datasets. At the population level, we calculated *D*_ps_, *F*_st_, and Nei's *D*_a_. These additional dissimilarity indices were chosen to allow direct discussion with the results of other similar studies (Luximon et al., 2014; Prunier et al., 2013). We then calculated correlations among the different genetic distance indices. Previous research has reported statistically significant pairwise *G*'_ST_ values across a portion of this area, with an overall *G*'_ST_ value of 0.246 (Goldberg & Waits, 2010a).

Our first test was to compare the influence of sampling schemes on genetic structure at the 18 sites where PSS occurred. For the PSS approach, we calculated *D*_ps_ between sites using the R package PopGenReport (Adamack & Gruber, 2014; R Core Team, 2015). This was done by calculating all pairwise proportions among all individuals and then averaging these matrices for each population pair. For the ISS approach, *D*_ps_ was calculated for each set of representative individual(s) (1–11 individuals) from each of the 18 sites using the propShared function in the R package PopGenReport (Adamack & Gruber, 2014; R Core Team, 2015). When the number of individuals was >1, we again calculated all pairwise proportions of shared alleles between individuals and then averaged all of these matrices between population pairs. In other words, in the cases of a single individual, we used proportion of shared alleles at each population, so the distance matrix was NxN which was equal to PxP with a single individual. For all sets beyond one individual per population, we used PxP, where we averaged the proportion of shared alleles among individuals. We simulated the ISS approach at both sampling densities by bootstrapping without replacement to create 100 datasets of representative individual(s). To compare the influence of sampling density on landscape genetic inference, we compared ISS at 18 sites to ISS at all 40 sites. We repeated the proportion of shared alleles calculation (ISS approach) for the 40 sites and compared with proportion of shared alleles for 18 sites as calculated above. For PASS, we calculated proportion of shared alleles using all available samples among all sampled sites.

### 2.2 Landscape variables and models

We examined how sampling scheme influenced landscape genetic inference using resistance analysis evaluated by information criterion metrics. Land cover (from Pocewicz et al., 2008) was split into separate rasters at a resolution of 15 m^2^: water, shrub, low‐density (LD) forest, high‐density (HD) forest, development, barren, agriculture, and grassland (Figure 1). Each raster was parameterized with two denoting raster cells containing the respective land cover type and one denoting any other land cover type. Riparian cover (from Redistricting Census 2000, U.S. Dept. of Commerce, Bureau of the Census, Geography Division) was parameterized this same way. Solar radiation and slope rasters were parameterized with their raw values. We calculated resistance surfaces for landscape variables (e.g., land cover type, solar radiation, and slope) by running each parameterized raster in CIRCUITSCAPE 4.0 (Shah & McRae, 2008), with a node file containing the 40 sampling locations. CIRCUITSCAPE models connectivity across the landscape by using circuit theory to calculate resistance between two points. We used maximum‐likelihood population‐effects (MLPE) models, where we ran linear mixed models with landscape resistance surfaces as predictor variables (Van Strien, Keller, & Holderegger, 2012). MLPE models address problems of nonindependence between pairwise comparisons of distance matrices through the use of random effects. In addition, to explicitly test for IBD, we created one model using a uniform (all cells equal one) resistance layer. We checked for multicollinearity using 18 and 40 sites, and iteratively removed the variable with the highest score until the model had a VIF threshold of <4 which has been suggested by other researchers (Table 2, Hair, Anderson, Tatham, & Black, 1998). Models were tested by a priori hypotheses, such as human impact (agriculture and development), energetic movement constraints (solar and slope), and undisturbed habitat (forest and grasslands) would influence frog movement. However, we did not optimize resistance values due to potential bias when relative impacts between pairs of variables are not explicitly known, such as differences between high‐ and low‐density forests. AICc and BIC were calculated from maximum‐likelihood (ML) estimations of the linear mixed models using the *lme4* package in R (Bates & Maechler, 2009). There are potential issues with AIC for models fit with restricted maximum‐likelihood REML when fixed effects are not the same across models being evaluated; however, information criterion ranking with MLPE models is found in the literature for model selection with REML under some circumstances, and we therefore used REML as well (Gurka, 2006; Row, Knick, Oyler‐McCanse, Lougheed, & Fedy, 2017). We then used AICc and ∆AICc to rank the likelihood of the 17 landscape hypotheses. For the ISS datasets, we also ranked each model based on frequency of high level of support for each bootstrapped dataset, using a threshold of ∆AICc < 2, to determine the number of times each model was competitive across replicate datasets. We conducted these analyses for the four sampling scheme datasets: ISS‐18, PSS, ISS‐40, and PASS.

**Table 2:** Variance inflation factor (VIF) results of fullest model during check for multicollinearity of landscape variables in northern Idaho at sample locations of Columbia spotted frogs (*Rana luteiventris*). VIF was calculated at each sampling density, 18 and 40 sites. Variables were removed iteratively based on the variable with the highest VIF score, until VIF score was lower than 4 for full model

	VIF Score	
	18 Sites	40 Sites
Shrub/Clear‐cut	2.77	2.58
Grass	1.25	1.10
Forest low density	1.86	1.34
Slope	1.99	2.59

## 3 RESULTS

Mean number of alleles per locus for the 18 populations was 4.410 ± 0.942 *SD* (Table 3). After Bonferroni correction, 2 out of 143 Hardy–Weinberg tests were significant at *p* < 0.05. Linkage disequilibrium was detected between 0 and 3 pairs of loci within each population, with 14 populations having no support for linkage disequilibrium at all loci. All loci were retained in the analysis because only one pair of loci showed linkage in more than one population. Rp3 and SFC139 showed linkage disequilibrium only in 4 of 18 populations; Rp3 and SFC139 were also found to be weakly linked in other studies of this species (Funk et al., 2005; Goldberg & Waits, 2010a). The chromosomal locations of these loci are unknown. Individual and population data can be found on DRYAD (https://doi.org/10.5061/dryad.1nq73).

**Table 3:** Population genetic analyses of Columbia spotted frog (*Rana luteiventris*) populations in northern Idaho, USA. We completed analyses on populations where a minimum of 11 individuals were collected. Eight microsatellite loci were used. *N* = samples size, A = total number of alleles across all microsatellite loci, uHe = unbiased expected heterozygosity, ML = number of monomorphic loci, LD = number of pairwise loci failed linkage disequilibrium test, HWE = number of loci failing Hardy–Weinberg equilibrium test. Statistical significance threshold *p* < 0.05 with Bonferroni correction, AR = average allelic richness, PA = mean number of private alleles across all loci

Pop #	*N*	A	uHe	ML	LD	HWE	AR	PA
1	15	34	0.604	0	1	0	4.03	0.500
2	21	40	0.614	0	3	0	4.40	0.125
3	11	42	0.588	0	0	0	5.25	0.125
4	11	30	0.558	0	0	0	3.75	0.250
5	15	39	0.669	0	0	0	4.57	0.375
6	16	26	0.526	0	1	0	3.12	0.000
7	14	32	0.472	1	0	0	3.75	0.000
8	11	32	0.636	0	0	0	4.00	0.000
9	11	28	0.500	1	0	0	3.50	0.000
10	25	46	0.664	0	0	0	4.87	0.000
11	18	46	0.670	0	0	0	4.94	0.500
12	14	40	0.621	0	0	1	4.76	0.000
13	16	23	0.381	2	1	0	2.68	0.000
14	20	38	0.553	0	0	0	4.29	0.000
15	12	40	0.719	0	0	0	4.93	0.000
16	17	41	0.625	0	0	0	4.93	0.000
17	11	20	0.494	0	1	0	2.63	0.000
18	18	44	0.656	0	0	1	4.89	0.250

**Figure 2:**
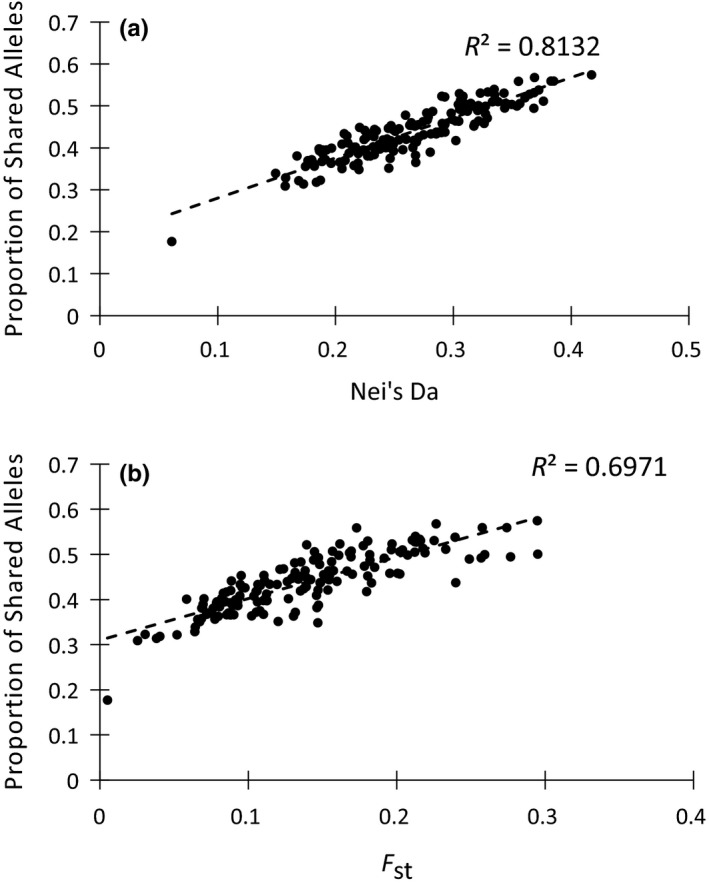
Correlation between proportion of shared alleles and Nei's *D*_a_ (a) and proportion of shared alleles and *F*_st_ (b) using sites with 11 or more individuals

Genetic distance dissimilarity indices were highly correlated using both the individual and populations sampling scheme datasets. With the individual sampling scheme dataset, Bray–Curtis percentage dissimilarity was equal to the inverse value of proportion of shared alleles (*D*_ps_). With the population sampling scheme dataset, the *R* ^2^ value for proportion of shared alleles versus Nei's *D*_a_ and *F*_st_ was 0.81 and 0.70, respectively (Figure 2). Because of these high correlations, we used the proportion of shared alleles metric in all downstream analyses.

Model support varied among the individual sampling scheme (ISS), population‐level sampling scheme (PSS), and proportion available sampling scheme (PASS). The population‐level dataset (PSS) had strong support for the low‐density forest cover model, while 48% of bootstrapped ISS datasets included this cover type in the set of models with more limited support. Using ML, all models comprised of a single landscape variable had support in greater than one‐fifth of the ISS replicate runs (Table 4). In the ISS‐18, ISS‐40, and PASS datasets, the fullest model and the model including low‐density forest, high‐density forest, and grass had the most support. The shrub/clear‐cut model also had support in the ISS‐40 dataset. However, for the PSS dataset, the highest level of evidence was present for the same model: low‐density forest cover, which was also frequently supported in the other datasets. BIC often supported models with fewer variables than AICc (Table 5). High‐density forest and slope were linearly correlated (*r* = 0.77), while each had a lower linear correlation with low‐density forest (ca. *r* = 0.45; Table 6). Top models were different when REML was used (AIC Table 7, BIC Table 8). Overall, model fit was similar across datasets. Plots of top performing models indicated homoscedasticity of residuals. Root‐mean‐square error for top performing models was <5% of the potential range of the dependent variable.

**Table 4:** Parameters and information theoretic (AIC) results for models of genetic distance (proportion of shared alleles) in north Idaho, USA, for the Columbia spotted frog (F*Rana luteiventris*) using maximum likelihood (ML). Parameters are land cover of low‐density forest (forestld), high‐density forest (foresthd), agriculture (ag), shrub/clear‐cut (shrub), human development (dev), grassland (grass), distance (distance), solar radiation (solar). Individual sampling scheme (ISS) results are reported as average ∆AICc of 100 bootstrapped replicates ± standard error (SE), with the number of times a model was competing (∆AICc < 2) reported in Num. Comp. Bold values indicate the top competing models (average AICc weight > 0.10). Population sampling scheme (PSS) represents sampling with a minimum of 11 individuals being collected per site. Proportion available sampling scheme (PASS) represents sampling with all individuals collected, regardless of number of individuals collected per site

Model	*k*	ISS, 18 sites			PSS, 18 sites		ISS, 40 sites			PASS, 40 sites	
		∆AICc	AICc Weight	Num. Comp.	∆AICc	AICc Weight	∆AICc	AICc Weight	Num. Comp.	∆AICc	AICc Weight
Grass + forest ld + forest hd + slope	7	**2.97**	**0.13 ± 0.00**	**36**	5.50	0.02	**3.77**	**0.24 ± 0.01**	**88**	**1.85**	**0.28**
Forest ld + forest hd + grass	6	**2.48**	**0.16 ± 0.00**	**50**	3.35	0.07	**7.04**	**0.10 ± 0.01**	**44**	**0.00**	**0.71**
Ag + dev	5	6.11	0.03 ± 0.00	11	9.14	0.00	**7.79**	**0.13 ± 0.02**	**54**	22.13	0.00
Solar + slope	5	4.39	0.06 ± 0.00	21	8.62	0.00	8.68	0.04 ± 0.01	20	18.94	0.00
Forest ld + dev	5	4.80	0.04 ± 0.00	20	**1.77**	**0.15**	8.99	0.06 ± 0.01	16	21.83	0.00
Solar + forest ld	5	5.18	0.05 ± 0.00	26	**1.61**	**0.16**	9.59	0.03 ± 0.01	4	21.73	0.00
Forest ld + shrub/cc	5	3.91	0.06 ± 0.00	28	**1.30**	**0.19**	**7.66**	**0.10 ± 0.01**	**42**	21.76	0.00
Ag	4	5.09	0.04 ± 0.00	29	10.85	0.00	12.05	0.02 ± 0.00	20	23.58	0.00
Forest hd	4	3.69	0.08 ± 0.00	42	11.73	0.00	10.57	0.05 ± 0.02	38	11.98	0.00
Grass	4	7.00	0.03 ± 0.00	21	15.39	0.00	14.35	0.02 ± 0.01	18	36.25	0.00
Slope	4	3.77	0.09 ± 0.00	44	18.04	0.00	8.35	0.06 ± 0.03	38	17.45	0.00
Dev	4	5.37	0.04 ± 0.00	29	7.51	0.01	10.07	0.04 ± 0.01	32	20.70	0.00
Riparian	4	5.91	0.03 ± 0.00	22	5.97	0.02	11.66	0.02 ± 0.00	12	21.65	0.00
Solar	4	5.96	0.03 ± 0.00	25	7.63	0.01	10.86	0.03 ± 0.00	24	20.32	0.00
Distance	4	5.60	0.03 ± 0.00	24	7.62	0.01	11.47	0.02 ± 0.00	20	22.87	0.00
Forest ld	4	6.28	0.04 ± 0.00	26	**0.00**	**0.36**	9.76	0.03 ± 0.01	26	19.99	0.00
Shrub/cc	4	5.00	0.04 ± 0.00	32	9.21	0.00	13.01	0.02 ± 0.00	14	24.82	0.00

**Table 5:** Parameters and information theoretic (BIC) results for models of genetic distance (proportion of shared alleles) in north Idaho, USA, for the Columbia spotted frog (*Rana luteiventris*) using maximum likelihood (ML). Parameters are land cover of low‐density forest (forestld), high‐density forest (foresthd), agriculture (ag), shrub/clear‐cut (shrub), human development (dev), grassland (grass), distance (distance), solar radiation (solar). Individual sampling scheme (ISS) results are reported as average BIC of 100 bootstrapped replicates ± standard error (*SE*), with the number of times a model was competing (∆BIC < 2) reported in Num. Comp. Bold values indicate the top competing models (average BIC weight > 0.10). Population sampling scheme (PSS) represents sampling with a minimum of 11 individuals being collected per site. Proportion available sampling scheme (PASS) represents sampling with all individuals collected, regardless of number of individuals collected per site

Model	*k*	ISS, 18 sites		PSS, 18 sites	ISS, 40 sites		PASS, 40 sites
		BIC Weight	Num. Comp.	BIC Weight	BIC Weight	Num. Comp.	BIC Weight
Grass + forest ld + forest hd + slope	7	0.01 ± 0.00	1	0.00	0.04 ± 0.01	10	0.03
Forest ld + forest hd + grass	6	0.05 ± 0.01	14	0.01	0.04 ± 0.01	9	**0.77**
Ag + dev	5	0.01 ± 0.00	1	0.00	0.09 ± 0.02	18	0.00
Solar + slope	5	0.04 ± 0.01	9	0.00	0.02 ± 0.01	2	0.00
Forest ld + dev	5	0.02 ± 0.00	1	0.07	0.03 ± 0.01	7	0.00
Solar + forest ld	5	0.04 ± 0.01	7	0.07	0.02 ± 0.01	4	0.00
Forest ld + shrub/cc	5	0.05 ± 0.01	9	0.08	0.09 ± 0.02	16	0.00
Ag	4	0.07 ± 0.00	15	0.00	0.04 ± 0.00	6	0.00
Forest hd	4	**0.15 ± 0.02**	**51**	0.00	**0.10 ± 0.02**	**23**	**0.17**
Grass	4	0.05 ± 0.01	18	0.00	0.03 ± 0.01	9	0.00
Slope	4	**0.15 ± 0.02**	**47**	0.00	**0.19 ± 0.02**	**44**	0.01
Dev	4	0.06 ± 0.00	15	0.02	0.08 ± 0.01	26	0.00
Riparian	4	0.05 ± 0.00	2	0.03	0.03 ± 0.00	1	0.00
Solar	4	0.05 ± 0.00	8	0.01	0.05 ± 0.00	16	0.00
Distance	4	0.05 ± 0.00	2	0.01	0.04 ± 0.00	9	0.00
Forest ld	4	0.08 ± 0.01	22	**0.68**	0.08 ± 0.01	24	0.00
Shrub/cc	4	0.07 ± 0.00	16	0.01	0.03 ± 0.00	5	0.00

**Table 6:** Correlation matrix results for land cover parameters of low‐density forest (forestld), high‐density forest (foresthd), agriculture (ag), shrub/clear‐cut (shrub), human development (dev), grassland (grass), distance (distance), solar radiation (solar) using all 40 populations of Columbia spotted frogs (*Rana luteiventris*) across northern Idaho, USA

	Forest ld	Shrub/cc	Distance	Slope	Forest hd	Ag	Dev	Solar	Grass	Riparian
Forest ld	1.000									
Shrub/cc	0.553	1.000								
Distance	0.673	0.889	1.000							
Slope	0.466	0.560	0.632	1.000						
Forest hd	0.456	0.702	0.744	0.773	1.000					
Ag	0.689	0.807	0.781	0.624	0.791	1.000				
Dev	0.662	0.867	0.988	0.642	0.712	0.762	1.000			
Solar	0.680	0.857	0.977	0.574	0.708	0.782	0.972	1.000		
Grass	0.221	0.526	0.661	0.174	0.207	0.460	0.661	0.674	1.000	
Riparian	0.659	0.818	0.963	0.554	0.677	0.721	0.949	0.966	0.678	1.000

**Table 7:** Parameters and information theoretic (AIC) results for models of genetic distance (proportion of shared alleles) in north Idaho, USA for the Columbia spotted frog (*Rana luteiventris*) using restricted maximum likelihood (REML). Parameters are land cover of low‐density forest (forestld), high‐density forest (foresthd), agriculture (ag), shrub/clearcut (shrub), human development (dev), grassland (grass), distance (distance), solar radiation (solar). Individual Sampling (ISS) results are reported as average AICc of 100 bootstrapped replicates +/‐ standard error (SE), with the number of times a model was competing (∆AICc < 2) reported in Num. Comp. Bold values indicate the top competing models (average AICc weight > 0.10). Population sampling scheme (PSS) represents sampling with a minimum of 11 individuals being collected per site. Proportion available sampling scheme (PASS) represents sampling with all individuals collected, regardless of number of individuals collected per site

		ISS, 18 sites			PSS, 18 sites		ISS, 40 sites			PASS, 40 sites	
Model	k	∆AICc	AICc Weight	Num. Comp.	∆AICc	AICc Weight	∆AICc	AICc Weight	Num. Comp.	∆AICc	AICc Weight
grass + forest ld + forest hd + slope	7	22.01	0.00 +/− 0.00	0	27.51	0.00	24.00	0.01 +/− 0.01	1	14.66	0.00
forest ld + forest hd + grass	6	14.91	0.00 +/− 0.00	0	18.66	0.00	19.63	0.01 +/− 0.01	2	5.12	0.06
ag + dev	5	10.46	0.00 +/− 0.00	0	15.84	0.00	10.94	0.06 +/− 0.02	8	17.87	0.00
solar + slope	5	9.79	0.01 +/− 0.01	1	16.69	0.00	12.57	0.01 +/− 0.01	1	15.48	0.00
forest ld + dev	5	9.70	0.01 +/− 0.00	0	9.17	0.01	12.78	0.02 +/− 0.01	3	18.20	0.00
solar + forest ld	5	10.04	0.01 +/− 0.01	1	9.00	0.01	13.37	0.01 +/− 0.01	2	18.08	0.00
forest ld + shrub/cc	5	8.84	0.02 +/− 0.01	4	8.83	0.01	11.48	0.05 +/− 0.01	8	18.06	0.00
ag	4	3.23	0.08 +/− 0.00	43	10.89	0.00	7.63	0.04 +/− 0.00	15	11.88	0.00
forest hd	4	**1.83**	**0.18 +/− 0.02**	**69**	11.74	0.00	**5.87**	**0.12 +/− 0.02**	**31**	**0.00**	**0.82**
grass	4	4.99	0.07 +/− 0.01	33	15.23	0.00	9.92	0.04 +/− 0.01	15	24.53	0.00
slope	4	**1.72**	**0.20 +/− 0.02**	**68**	17.88	0.00	**3.29**	**0.29 +/− 0.03**	**58**	4.94	0.07
dev	4	3.66	0.07 +/− 0.00	36	7.77	0.02	5.94	0.09 +/− 0.01	23	9.32	0.01
riparian	4	4.11	0.06 +/− 0.00	36	6.26	0.04	7.55	0.03 +/− 0.00	7	10.31	0.00
solar	4	4.26	0.06 +/− 0.00	34	7.95	0.02	6.79	0.05 +/− 0.00	16	9.01	0.01
distance	4	3.92	0.06 +/− 0.00	31	7.92	0.02	7.36	0.04 +/− 0.00	13	11.52	0.00
forest ld	4	**4.34**	**0.10 +/− 0.01**	**48**	**0.00**	**0.87**	**5.37**	**0.11 +/− 0.01**	**28**	8.25	0.01
shrub/cc	4	3.30	0.08 +/− 0.00	44	9.43	0.01	8.60	0.03 +/− 0.00	11	13.14	0.00

**Table 8:** Parameters and information theoretic (BIC) results for models of genetic distance (proportion of shared alleles) in north Idaho, USA, for the Columbia spotted frog (*Rana luteiventris*) using restricted maximum likelihood (REML). Parameters are land cover of low‐density forest (forestld), high‐density forest (foresthd), agriculture (ag), shrub/clear‐cut (shrub), human development (dev), grassland (grass), distance (distance), solar radiation (solar). Individual sampling scheme (ISS) results are reported as average BIC of 100 bootstrapped replicates ± standard error (*SE*), with the number of times a model was competing (∆BIC < 2) reported in Num. Comp. Bold values indicate the top competing models (average BIC weight > 0.10). Population sampling scheme (PSS) represents sampling with a minimum of 11 individuals being collected per site. Proportion available sampling scheme (PASS) represents sampling with all individuals collected, regardless of number of individuals collected per site

Model	*k*	ISS, 18 sites		PSS, 18 sites	ISS, 40 sites		PASS, 40 sites
		BIC Weight	Num. Comp.	BIC Weight	BIC Weight	Num. Comp.	BIC Weight
Grass + forest ld + forest hd + slope	7	0.00 ± 0.00	0	0.00	0.00 ± 0.00	0	0.00
Forest ld + forest hd + grass	6	0.00 ± 0.00	0	0.00	0.00 ± 0.00	0	0.00
Ag + dev	5	0.00 ± 0.00	0	0.00	0.03 ± 0.02	5	0.00
Solar + slope	5	0.01 ± 0.01	1	0.00	0.00 ± 0.00	1	0.00
Forest ld + dev	5	0.00 ± 0.00	1	0.00	0.00 ± 0.00	2	0.00
Solar + forest ld	5	0.01 ± 0.00	1	0.00	0.00 ± 0.00	1	0.00
Forest ld + shrub/cc	5	0.01 ± 0.01	2	0.00	0.01 ± 0.00	5	0.00
Ag	4	0.08 ± 0.00	23	0.00	0.04 ± 0.00	8	0.00
Forest hd	4	**0.18 ± 0.02**	**60**	0.00	**0.13 ± 0.02**	**35**	**0.88**
Grass	4	0.07 ± 0.01	25	0.00	0.05 ± 0.01	14	0.00
Slope	4	**0.21 ± 0.02**	**65**	0.00	**0.33 ± 0.03**	**66**	0.07
Dev	4	0.07 ± 0.00	16	0.02	0.09 ± 0.01	25	0.01
Riparian	4	0.06 ± 0.00	3	0.04	0.03 ± 0.00	0	0.01
Solar	4	0.06 ± 0.00	8	0.02	0.05 ± 0.00	13	0.01
Distance	4	0.06 ± 0.00	2	0.02	0.04 ± 0.00	8	0.00
Forest ld	4	**0.11 ± 0.01**	**41**	**0.89**	**0.14 ± 0.02**	**42**	0.01
Shrub/cc	4	0.08 ± 0.00	19	0.01	0.03 ± 0.00	6	0.00

**Figure 3:**
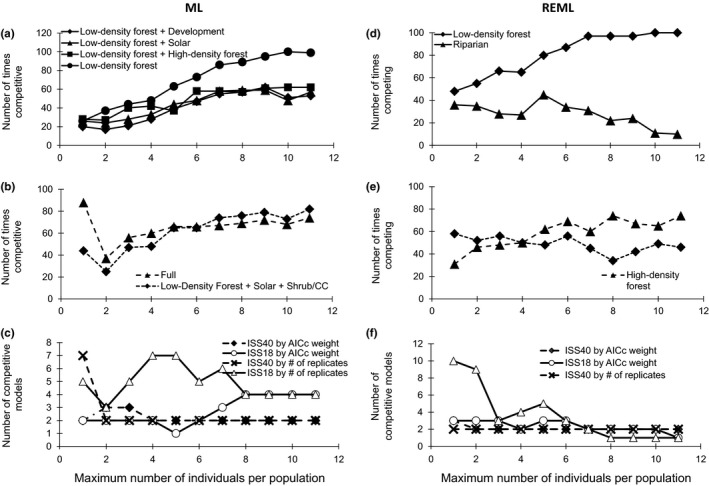
Number of times model was competing (∆AICc < 2) out of 100 replicate datasets and number of competing models for varying number of individuals randomly sampled from full dataset of *Rana luteiventris* in the Palouse region near Moscow, ID. (a, d) Varying number of individuals at sites where minimum number of individuals allowed for population‐level genetic distance calculations (*N* = 18). Low‐density forest was the top model when using all individuals for the PSS dataset (average AICc weight = 0.87) while riparian was until 8 individuals were used when using REML. Forest low density + development or solar or forest high‐density or by itself were most competitive in the PSS dataset when ML (average AICc weight = 0.15, 0.16, 0.19, and 0.36, respectively). (b, e) Varying number of individuals at all sites sampled (*N* = 40). High‐density forest was the top model using all individuals in the PASS dataset (AICc weight = 0.82); slope was correlated with high‐density forest and was competitive across all ISS models when using the 40‐site dataset with REML. The fullest model and forest low density + shrub/clear‐cut + grass were most competitive when using ML (average AICc weight = 0.28 and 0.71, respectively). (c, f) Number of competing models as number of individuals was increased. Competitive threshold for average AICc weight was 0.1, and threshold for number of replicates out of 100 was 30 replicates

When number of individuals per site was increased, ISS converged with the sampling scheme of the same density and extent (PSS and PASS for the ISS at 18 and 40 sites, respectively). When nine individuals were included, ISS at 18 sites had >90% of datasets having model support agreeing with the one of the top PSS models, of low‐density forest land cover being competitive (Figure 3a). ISS at 40 sites converged with PASS, with the two top models supported although never at greater than 90% (Figure 3b). Total number of competitive models as number of individuals was increased showed a pattern of decreasing number of competitive models as number of individuals increased, except for the ISS‐18 models, which showed an increase then decrease in number of competitive models (Figure 3c). ISS at 40 sites supported 1 to 2 models based on both average AICc weight and number of times supported across replicates once more than a single individual was used (Figure 3c). Pattern of the number of times a model was competitive was similar when REML was used, and additional individuals were added (Figure 3d, e). ISS at 18 sites had a roughly linear decline in the number of supported models across replicates as number of samples per site when increased from 1 to 9 with a large proportion of the models being supported when a low number of individuals were used (Figure 3f). This pattern was not seen when counting number of supported models based on average AICc weight, where supported models varied from 3 to 2.

## 4 DISCUSSION

Landscape genetics is a powerful method for evaluating functional connectivity (Manel et al., 2003, Holderegger & Wagner, 2006, Manel & Holderegger 2013), but appropriate sampling strategies and schemes can be difficult to determine and apply (Manel et al., 2003; Segelbacher et al., 2010). We found that inferences differed between individual and population sampling schemes when we compared the two datasets at 18 sites. This pattern for ISS‐18 was likely due to a lack of statistical power. At 40 sites, the models supported by PASS were also supported by the ISS at 40 sites, but the support for the PASS top model was not as strong as for the full dataset and additional models were supported with the ISS‐40 dataset. Support varied considerably within the ISS‐18 and ISS‐40 datasets as well. No model was supported more than 90 percent, and with most of the models being supported around 25 percent of the time. With increased numbers of individuals sampled, the ISS converged with PSS and PASS. Convergence occurred at nine individuals with ML, but seven individuals with REML. This indicates that small numbers of sampled individuals may be appropriate under certain circumstances, for example, stronger population structure or increased number of loci (Landguth et al., 2010, 2012; Prunier et al., 2013). However, the variation in model support suggests caution is important as mistaken inferences may be drawn if sample size is insufficient. The differences between REML and ML occurred at lower numbers of individuals per population, which highlights a potential methodological issue when moving to an ISS.

Prunier et al. (2013) found that the individual sampling scheme could, in most cases, have similar inferences of landscape connectivity when compared with the population sampling scheme, when moving to sampling three or four individuals, as opposed to a single individual, per population. In contrast, we found that more individuals were needed in our study to reach the same conclusions between sampling schemes regardless of statistical methods or number of populations. The empirical example of Prunier et al. (2013) had 78 populations where at least two alpine newts were captured for an ISS, with six populations meeting their PSS threshold of 20 individuals. This increased statistical power due to a higher number of sites for their evaluation of ISS than presented in our research may explain the differences in minimum number of individuals required to converge. Unlike the ISS at 18 sites, there was not a threshold for ISS at 40 sites as the number of samples approached the PASS. However, the top model from the PASS did have increased support as we added more individuals. This may be due to the inherent stability in the data for the PASS between replicates; at some of the 40 sites, only a single or few individuals were surveyed, so as the maximum number of individuals per site was increased, there was no change in the available genetic data for some of the sites. Because PASS has only one realization and the two competing models in the bootstrapped replicates had correlated variables, it is possible that PASS supported high‐density forest over slope by chance.

Comparisons between this research and similar past research highlight several general points about sampling schemes in landscape genetics. First, based on the results from Prunier et al. (2013) and the present study, the number of individuals needed to reach correct landscape genetic inference varies by system but may be somewhere between three and nine. Simulations using large numbers of single nucleotide polymorphism (SNP) loci have also found sample sizes of 4–6 may be appropriate for obtaining accurate estimates of *F*_st_ (Willing, Dreyer, & Oosterhout, 2012), and subsequent inferences from landscape genetics. This is much lower than the standard recommended value of 20–30 individuals per population (Storfer et al., 2007). It may be appropriate to lower target sample number goals based on these results. However, the number of individuals needed is going to be related to the amount of population differentiation (Kalinowski, 2005). By lowering the number of individuals per population, researchers will be able to increase either the spatial extent or sampling density of their studies or save valuable resources. In our system, shifting from 20 to 7 individuals per site would allow us to double the density or extent of future projects. However, both our study and Prunier et al. (2013) focused on amphibian species with easy to delineate breeding populations, and additional research is needed with more continuously distributed species. In general, sampling scheme may vary with the study system, and it may be most appropriate to evaluate the optimal sample size in situ. Researchers could achieve this using similar bootstrapping methods as presented here with empirical datasets.

Generally, we saw different top models per information criteria methods with ML and REML estimations. For the PSS, low‐density forest model was supported by both ML and REML, but with ML, three additional competing models were also supported, all of which included the low‐density forest variable when using AICc. This pattern of support for models with more variables with ML was also seen with PASS, where evidence weight was highest for the two models with the greatest number of variables with ML, but REML showed strong support for a single variable model, forest high‐density. As with PSS, this variable was present in the multivariable models supported with ML estimation. Top models for ISS‐18 and ISS‐40 with REML were also different, with three single variable models but with ML support was greatest for models with the most variables. This highlights potentially different inferences derived from model selection methodology depending on the estimation used, particularly when dealing with ISS. BIC results were more similar between ML and REML estimation, potentially due to the sample sizes we had available and the use of sample size corrected AIC. Because fixed effects vary among the models that were tested in these cases, ML would be most appropriate as opposed to REML (Verbeke & Molenberghs, 2000), although there are examples in the literature of using REML for MLPE (e.g., Emel & Storfer, 2014, Gurka, 2006, Row et al., 2017, Zancolli, Rodel, Steffan‐Dewenter, & Storfer, 2014). We provide both sets of results to highlight this aspect of model selection. Although limited by empirical data here, more extensive ML compared to REML testing with MLPE using simulations may be important to fully address best practices in model selection methodology.

This study identifies landscape features that impede gene flow for the Columbia spotted frog while also assessing the effects of different sampling strategies on landscape genetic inference. Low‐density forest was the most supported model with PSS using ML and at the smaller sampling extent and density when using REML, while high‐density forest was the most supported model at the increased sampling extent and density when using REML. In addition, low‐density and high‐density forest occurred in almost all of the supported models when ML was used. Results of low‐density forest and high‐density forest models indicate a reduction in gene flow when these land cover types occur between breeding populations. These results are similar to those previously found for this species at a finer scale in the southwestern portion of this study area, where forest was found to restrict gene flow (Goldberg & Waits, 2010a). However, Goldberg and Waits (2009) found breeding sites to be near low‐ and high‐density forest; specifically, proximity to low‐density forest was the most important land cover variable for the presence of breeding populations of this species. Together, these findings indicate the important influence that forests have on Columbia spotted frogs in this region, and the difference between habitat requirements and land cover contributions to functional connectivity.

Landscape features may influence gene flow differently across a species range depending on range‐wide variation in interactions with climate and other variables. In this study area, slope was not supported outside of a correlation with high‐density forest, but in more mountainous regions, topographic features were supported as an important influence on functional connectivity for this species (Funk et al., 2005; Murphy, Dezzani, Pilliod, & Storfer, 2010). The range of variation of interest may be key to explaining observed differences among study regions, which may explain differences in inferences of range‐wide connectivity (Cushman & Landguth, 2010; Short Bull et al., 2011). Although not tested in this study, there is evidence that other at‐site abiotic and biotic factors, such as frost‐free period, presence of predatory fish, and site productivity, are also important for this species (Murphy et al., 2010).

It is important to highlight the limitations of this research as it uses empirical data on a single species, the Columbia spotted frog. This species has clear population boundaries, and further research into this sampling question could benefit from a focus on species that are more continuous distributed or at lower densities across the landscape. For example, Luximon et al. (2014) found that ISS was more appropriate with continuously distributed species in simulation. The minimum number needed to reach similar results to a population sampling scheme may depend on the degree of population structure and distribution. Using an individual sampling scheme approach may reduce concerns of defining populations for landscape genetic analyses when a priori population delineations do not exist. Of particular concern, if genetic clustering algorithms are used, is that erroneous population delineations may occur depending on the assumptions and levels of migration within the system. Erroneous delineation could lead to incorrect downstream landscape genetic inferences, as inappropriate grouping of populations may drastically change calculated genetic distances (Schwartz & McKelvey, 2008). Detecting correct population structure can also be influenced by uneven sampling sizes; uneven sampling can lead to an incorrect inference on the number of subpopulations (Puechmaille, 2016). Working with empirical data also meant that we were limited to treating the dataset with the greatest statistical power as truth, in contrast with simulation studies. It is possible that the results from the population sampling scheme do not reflect the true drivers of functional connectivity in this system; however, it represented our most‐informed dataset.

One additional concern may be that we used unweighted allele‐sharing genetic distances. This system has high levels of population structure (Goldberg & Waits, 2010a); because frequency‐weighted allele‐sharing metrics are often void of the grouping of the individuals (e.g., Greenbaum, Templeton, & Bar‐David, 2016), it may not be appropriate to apply them in cases with such patterns of isolation. Second, frequency‐weighted allele‐sharing metrics, such as Lynch and Ritland (1999), appear to be most appropriate when dealing only with levels of high relatedness (Van De Casteele, Galbusera, & Matthysen, 2001). Third, for some frequency‐weighted allele‐sharing genetic distance metrics, it is assumed frequency distributions are identical for all loci and mating was completely random, which is unlikely to be the case in anuran systems (Arak, 1988; Davies & Halliday, 1977; Howard, 1980; Reading, 2001).

The most appropriate sampling scheme in landscape genetics is still a question that needs further investigation, and will vary by system (Balkenhol *et al*. 2015, Segelbacher et al., 2010, Storfer et al., 2007). Increased statistical power is obtained by increasing individuals sampled (Prunier et al., 2013), and so increasing sampling density or extent by adding more individuals or populations may result in similar conclusions; however, we observed increasing extent may not result in the same conclusion if the additional area encompasses different landscape patterns and processes. The question of increasing extent or sampling density in lieu of number of samples per site is something that needs to be explored through both simulations and additional empirical work. This is particularly true with the proposed PASS sampling scheme, which allowed us to increase the study extent as opposed to removing sites completely due to not reaching the targeted number of individuals per population. In addition, research on the proportion available sampling scheme may help with experimental design where available number of individuals varies with site. Other systems and datasets may already be available to explore these questions, and simply incorporating the bootstrap methodology used here could allow for more robust inferences on sampling scheme by adding additional systems to test the observed thresholds.

## CONFLICT OF INTEREST

None declared.

## AUTHOR CONTRIBUTIONS

Travis Seaborn is the primary author, involved in computer coding, prepared final figures, table and article. Samantha Hauser coded, wrote, edited, and drafted figures and article. Lauren Konrade coded, wrote, and edited the article. Lisette Waits is secondary PI, designed the project, and edited the article. Caren Goldberg is the principal investigator, designed the project, coded the article, collected and processed the samples.

## Data Availability

Dataset for individual and population information: DRYAD entry https://doi.org/10.5061/dryad.1nq73.
